# Cathelicidin Insufficiency in Patients with Fatal Leptospirosis

**DOI:** 10.1371/journal.ppat.1005943

**Published:** 2016-11-03

**Authors:** Janet C. Lindow, Elsio A. Wunder, Stephen J. Popper, Jin-na Min, Praveen Mannam, Anup Srivastava, Yi Yao, Kathryn P. Hacker, Khadir Raddassi, Patty J. Lee, Ruth R. Montgomery, Albert C. Shaw, Jose E. Hagan, Guilherme C. Araújo, Nivison Nery, David A. Relman, Charles C. Kim, Mitermayer G. Reis, Albert I. Ko

**Affiliations:** 1 Department of Epidemiology of Microbial Diseases, Yale School of Public Health, New Haven, Connecticut, United States of America; 2 Centro de Pesquisas Gonçalo Moniz, Fundação Oswaldo Cruz, Ministério da Saúde, Salvador, Bahia, Brazil; 3 Department of Medicine, Stanford University School of Medicine, Stanford, California, United States of America; 4 Section of Pulmonary, Critical Care and Sleep Medicine, Yale University School of Medicine, New Haven, Connecticut, United States of America; 5 Section of Rheumatology, Department of Internal Medicine, Yale School of Medicine, New Haven, Connecticut, United States of America; 6 Department of Neurology, Yale School of Medicine, New Haven, Connecticut, United States of America; 7 Section of Infectious Diseases, Department of Internal Medicine, Yale School of Medicine, New Haven, Connecticut, United States of America; 8 Department of Microbiology and Immunology, Stanford University School of Medicine, Stanford, California, United States of America; Veterans Affairs Palo Alto Health Care System, Palo Alto, California, United States of America; 9 Division of Experimental Medicine, Department of Medicine, University of California, San Francisco, San Francisco, California, United States of America; University of Montana, UNITED STATES

## Abstract

Leptospirosis causes significant morbidity and mortality worldwide; however, the role of the host immune response in disease progression and high case fatality (>10–50%) is poorly understood. We conducted a multi-parameter investigation of patients with acute leptospirosis to identify mechanisms associated with case fatality. Whole blood transcriptional profiling of 16 hospitalized Brazilian patients with acute leptospirosis (13 survivors, 3 deceased) revealed fatal cases had lower expression of the antimicrobial peptide, cathelicidin, and chemokines, but more abundant pro-inflammatory cytokine receptors. In contrast, survivors generated strong adaptive immune signatures, including transcripts relevant to antigen presentation and immunoglobulin production. In an independent cohort (23 survivors, 22 deceased), fatal cases had higher bacterial loads (*P* = 0.0004) and lower anti-*Leptospira* antibody titers (*P* = 0.02) at the time of hospitalization, independent of the duration of illness. Low serum cathelicidin and RANTES levels during acute illness were independent risk factors for higher bacterial loads (*P* = 0.005) and death (*P* = 0.04), respectively. To investigate the mechanism of cathelicidin in patients surviving acute disease, we administered LL-37, the active peptide of cathelicidin, in a hamster model of lethal leptospirosis and found it significantly decreased bacterial loads and increased survival. Our findings indicate that the host immune response plays a central role in severe leptospirosis disease progression. While drawn from a limited study size, significant conclusions include that poor clinical outcomes are associated with high systemic bacterial loads, and a decreased antibody response. Furthermore, our data identified a key role for the antimicrobial peptide, cathelicidin, in mounting an effective bactericidal response against the pathogen, which represents a valuable new therapeutic approach for leptospirosis.

## Author Summary

Leptospirosis causes over one million cases and nearly 60,000 deaths annually. Infection with the spirochetal bacterium results in a spectrum of symptoms, ranging from mild febrile illness to life-threatening pulmonary hemorrhage syndrome and acute kidney injury. Despite leptospirosis being a leading cause of zoonotic morbidity worldwide, little is known about the human immune response to *Leptospira* infections, and less about the pathogenic mechanisms resulting in severe disease outcomes. Here, we used a systems biology approach to discover transcripts and immunoprofiles associated with case fatality. We identified new risk factors for high bacterial loads and fatal leptospirosis, including the antimicrobial peptide, cathelicidin, which we validated in an animal model. Cathelicidin therefore represents a potential novel treatment for severe cases of leptospirosis.

## Introduction

Pathogenic *Leptospira spp* cause life-threatening disease, primarily in the world’s most impoverished populations [1]. Leptospirosis is considered the most widespread zoonotic disease due to the large number of wild and domestic mammalian reservoirs [2] and causes an estimated 1.03 million infections and 59,000 deaths globally per year [3, 4]. In Brazil alone, epidemic outbreaks of leptospirosis in urban slum communities during seasonal periods of heavy rainfall account for more than 10,000 reported cases each year [5, 6]. Despite its widespread importance, development of a vaccine has been hampered by genetic and antigenic diversity in pathogenic *Leptospira*, which are comprised of ten species and >200 serovars. Humans are accidental hosts and acquire the disease through contact with water or soil contaminated with *Leptospira* excreted in the urine of reservoir hosts. During a systemic infection, clinical manifestations can range from a self-limiting febrile illness to Weil´s disease, the classic severe form with jaundice, acute renal failure and bleeding, or severe pulmonary hemorrhage syndrome (LPHS) [1, 7, 8]. Notably, case fatality rates from Weil’s disease and LPHS are >10% and 50%, respectively [7, 8, 9, 10].

At present, the factors contributing to disease progression and poor clinical outcomes in patients with leptospirosis are poorly understood. No studies to date have found associations between genetic differences in *Leptospira spp* and poor disease outcomes, suggesting other factors drive disease severity [11, 12]. The infecting inoculum dose may also affect patient outcomes, but these have been intrinsically difficult to measure and evaluate. Alternatively, differences in host factors, such as the immune response to bacteria, are known to contribute in general to the development of lung injury and septic shock, and may be relevant to severity of responses to *Leptospira* infection [13–16].

Several lines of evidence suggest that the pathology associated with severe disease, LPHS and Weil’s syndrome, is in part, immune-mediated. In the city of Salvador, Brazil, a single serovar, *L*. *interrogans* serovar Copenhageni, causes the full spectrum of disease, suggesting that strain-specific differences in pathogen virulence do not explain differences in disease outcome [7, 17–19]. Furthermore, patients with poor outcomes, such as fatality, have been shown to have altered cytokine responses, including elevated mRNA transcripts of IL-1α and its antagonist receptor, IL-1RA, higher serum levels of IL-10 and IL-6, and high ratios of IL-10:TNFα [20–24]. These cytokines are commonly associated with innate immune responses; however, such cytokine responses are largely uncharacterized in patients with leptospirosis, despite neutrophilia being a common disease characteristic, and the known protective or detrimental roles neutrophils play in other bacterial infections [25–27]. Potential roles for T cells and endothelial cells in poor disease outcomes have also been described, but these remain less well validated in patient investigations [28]. While antibodies appear protective in experimental animal models of leptospirosis [29–32], definitive roles for B and T cells in the resolution or exacerbation of human *Leptospira* infections remain largely uncharacterized.

A better understanding of the human response to *Leptospira* infection could discern likely pathogenic processes involved in disease development. To identify features of disease response associated with death or survival, we conducted an in-depth multi-parameter analysis of immune responses during the acute phase of leptospirosis in a well-characterized cohort of hospitalized patients, including assessment of transcriptional profiles, serum components, and immune cell abundances. This work contributes to our understanding of immunopathogenic processes that affect disease outcome and identifies novel approaches to therapeutic intervention for leptospirosis.

## Results

### Specific Clinical and Laboratory Features Define Deceased Leptospirosis Patients

To identify host factors contributing to fatality, we enrolled 16 patients hospitalized with acute leptospirosis (13 survivors, 3 fatal cases) and 4 healthy community volunteers for in-depth characterization of clinical course and immune responses. Table 1 describes the patient characteristics for biochemical and clinical values during hospitalization for fatal and nonfatal cases. As noted in other studies, we observed that fatal cases had significantly elevated percentages of neutrophils as well as lower minimum hematocrit and percent lymphocytes in peripheral blood [23, 26]. We also found that acute phase anti-*Leptospira* agglutinating antibody titers were lower and *Leptospira* loads trended higher in the deceased group. Of the outcomes measured, we determined that only acute lung injury was more frequently associated with the deceased group. We found no differences in days of symptoms prior to admission (*P* = 0.26), age (*P* = 0.42), gender (*P* = 0.35), or days of symptoms prior to microarray sampling (*P* = 0.33) between survivors (8.4 ± 1.9 days) and nonsurvivors (6.7 ± 2.3 days). Thus, our patient cohort is representative of disease outcomes common to leptospirosis in Brazilian patients.

**Table 1:** Characteristics of leptospirosis patients during hospitalization.

CHARACTERISTICS	SURVIVORS		DEATHS			
	N	Median (IQR) or N (%)	N	Median (IQR)or N (%)	*P*-value
Age	13	29.0 (22.5–39.0)	3	32.0 (32.0–36.0)	-
Gender (Male)	13	12 (92%)	3	2 (67%)	-
CLINICAL PRESENTATION					
Days of illness^a^	13	7.0 (5.5–7.5)	3	5.0 (4.0–7.0)	-
Fever	13	13 (100%)	3	3 (100%)	-
Jaundice	13	9 (69%)	3	1 (33%)	-
Oliguria	13	1 (8%)	3	1 (33%)	-
Dyspnea^b^	13	2 (15%)	3	1 (33%)	-
CLINICAL LABORATORY					
Hematocrit (%)	13	32.4 (6.9)	3	26.7 (4.7)	0.05
Leukocytes (1000/μL)^d^	13	13.7 (9.5–19.8)	3	17.0 (11.4–72.6)	-
% Neutrophils^d^	13	78.0 (74.5–85.5)	3	94.0 (89.0–95.0)	0.01
% Lymphocytes^c^	13	13.0 (7.5–18.5)	3	4.0 (2.0–7.0)	0.02
Platelets (1000/μL)^c^	13	97.0 (38.0–167.0)	3	26.0 (15.0–38.0)	0.06
Serum creatinine (mg/dL)^d^	13	2.4 (1.7–4.0)	3	3.6 (2.7–8.8)	-
Blood urea nitrogen (mg/dL)^d^	13	91.0 (45.5–103.5)	3	63.0 (53.0–295.0)	-
Serum potassium (meq/L)^d^	13	4.3 (3.8–4.8)	3	5.0 (3.7–6.8)	-
Serum bilirubin (mg/dL)^d^					
Direct	11	6.5 (1.0–19.6)	2	8.4 (4.3–12.4)	-
Indirect	11	1.5 (0.7–6.0)	2	2.4 (1.2–3.6)	-
HOSPITAL OUTCOMES					
Hospitalization days	13	7.0 (6.0–8.5)	3	2.0 (1.0–12.0)	-
ICU admission	13	2 (15%)	3	1 (33%)	-
Dialysis	13	1 (8%)	3	1 (33%)	-
Oliguria^e^	13	2 (15%)	3	1 (33%)	-
Mechanical ventilation	13	0 (0%)	3	2 (67%)	0.030
Pulmonary hemorrhage^f^	13	0 (0%)	3	1 (33%)	-
Acute lung injury^g^	13	2 (15%)	3	3 (100%)	0.018
Respiratory failure^h^	13	0 (0%)	3	3 (100%)	0.002
LABORATORY DIAGNOSIS					
Agglutinating antibody titers					
Acute-phase	13	800 (250–2400)	3	0 (0)	0.03
Convalescent-phase	13	3200 (1600–6400)	0	N/A	N/A
*Leptospira* load (Geq/mL)^i^	11	0 (0–208)	3	14586 (0–20828)	0.052

^a^ Prior to hospital admission.

^b^ Maximum respiratory rate ≥ 38 breaths per minute during hospitalization.

^c^ Values represent minimum values during hospitalization.

^d^ Values represent maximum values during hospitalization.

^e^ Oliguria (<500mL urine/day) or anuria (<50mL urine/day) or patient received hemodialysis.

^f^ >250 mL blood in the lungs or large volume of blood in an endotracheal tube.

^g^ Mechanical ventilation, massive pulmonary hemorrhage, and/or maximum respiratory rate ≥ 38 breaths per minute during hospitalization.

^h^ Mechanical ventilation or massive pulmonary hemorrhage during hospitalization.

^i^ Geometric mean of *Leptospira* genomes/mL as determined by RT-qPCR.

### Strong Innate and Adaptive Immune Responses Distinguish Acute Disease from Convalescence and Healthy Volunteers

To delineate host responses important during acute leptospirosis, we performed a transcriptional analysis of whole blood comparing paired samples from acute disease and convalescence from 13 survivors and a single sample from four healthy Brazilian volunteers (S1 Text). As expected, many genes (1089 unique transcripts) were differentially expressed during acute infection relative to convalescence (S1A Fig). Of these, 363 transcripts increased in relative abundance during acute illness relative to convalescence, while 726 increased in convalescence relative to acute infection (S1 Table). To identify pathways associated with the acute phase of leptospirosis, we performed a functional analysis (DAVID) of all 1089 significant transcripts and found 40 significant (FDR < 0.01 and Benjamini <0.05) Gene Ontology terms (GO terms) enriched in acute versus convalescent comparisons (S2 Table) [33, 34]. These include categories such as “response to bacterium”, “defense response”, “antigen binding”, and 72 transcripts for immunoglobulin or immunoglobulin-like genes, which were enriched 2.0–7.9-fold during acute illness. Notably, no genes were significantly different between the convalescent and healthy volunteer groups, indicating that the immune state had returned to baseline 1–3 months following hospitalization (S1 Fig).

### Transcriptional Profiles Distinguished Survivors from Fatal Cases

**Fig 1:**
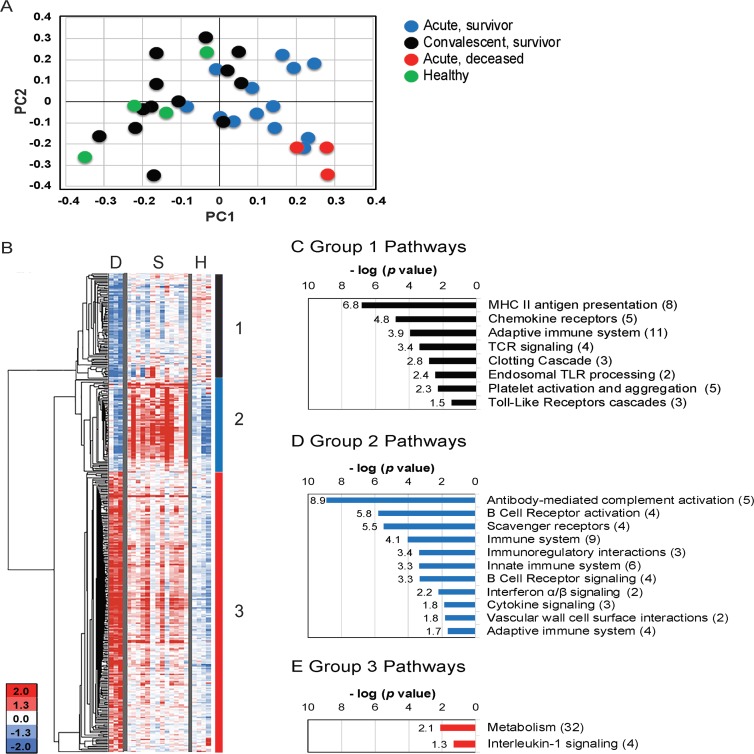
Transcriptional signatures associated with fatal cases. **(A)** PCA of all probes for 3 patient groups and healthy volunteers. **(B)** Heatmap depicting hierarchical clustering of 471 probes with differential expression during acute illness in 3 deceased patients (D) and 13 acute survivors (S). For comparison, the same transcripts for 4 healthy volunteers are shown (H). Blue indicates down-regulation and Red indicates up-regulation in log_2_. **(C-E)** Functional REACTOME pathways for 3 expression groups with negative log of p-values and number of genes in parentheses. In Groups 1 (black box) and 2 (blue box), transcripts were enriched in survivors vs fatal cases, while in Group 3 (red box), transcripts were enriched in fatal cases.

When we examined patterns of gene expression in the 16 cases using principal component analysis, the first principal component (PC1) (which explained 9.9% of the variance), separated the acute patient samples from paired convalescent and healthy volunteer samples (*P* = 0.0001) (Fig 1A). Strikingly, we found fatal acute disease profiles separated significantly from survivor profiles in principal component 2 (PC2) (7.6% variance; *P* = 0.014). These data support the hypothesis that host-derived factors are associated with fatal outcomes. We therefore directly compared the acute phase transcriptional profiles of 13 nonfatal and three fatal cases to identify specific gene expression changes associated with survival and death.

We identified 389 differentially expressed (DE) unique transcripts in deceased patients versus survivors (Fig 1B). We categorized the DE transcripts into three expression profile groups based on co-expression patterns after hierarchical clustering (Figs 1B and S1A). Groups 1 and 2 represent transcripts more abundant in nonfatal cases, with group 2 transcripts (92 unique genes) elevated during the acute phase of illness compared to convalescence, and Group 1 transcripts (76 unique genes) stable across nonfatal cases and not significantly different from convalescence (Fig 1B and S3 Table). Group 3 contains 221 transcripts with higher abundance in deceased patients compared to acute phase survivors or convalescents (Fig 1B and S3 Table).

Despite survivors presenting with varying disease severity during acute infection, only 27% (N = 105) of all significant transcripts from acute phase survivors (compared to convalescence) exhibited differential expression when compared with those of deceased patients (S1D Fig and S1 Table). Further, a majority of all the transcripts, elevated during acute infection in survivors, were not elevated during acute infection in deceased patients, suggesting a specific transcriptional alteration in fatal cases (S1D Fig).

### Fatal Cases Exhibited Decreased Transcription of Genes Involved in Chemotaxis, Coagulation, and Adaptive Immune Responses

**Fig 2:**
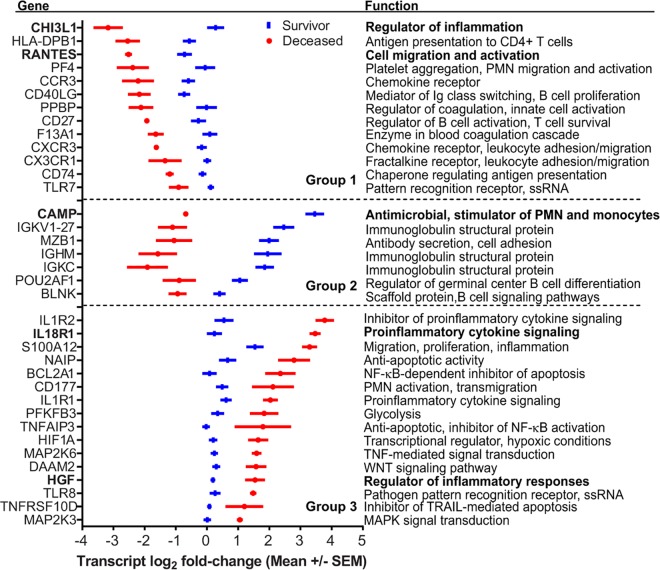
Specific transcripts associated with case fatality. Values are the average normalized log_2_ fold-change of signal intensities ± Standard Error of the Mean for select transcripts in Groups 1–3 described in Fig 1. The gene names are shown on the left and the functional annotation is shown on the right. Genes were selected based on their fold-change in Deceased vs Survivor (DvS) comparisons and had significant q values.

We performed functional enrichment analyses for transcripts more abundant in each of the three deceased vs survivor signature groups to discover molecular mechanisms that may have contributed to fatal disease outcomes (Fig 1C–1E; S2 and S4 Tables) [33, 34]. Within Group 1 transcripts, we identified 38 significant GO terms and 30 REACTOME pathways (Fig 1C; S2 and S4 Tables), the vast majority of which were related to immune function or coagulation. Of note, the chemokine *CCL5* (*RANTES*), important for recruitment of T cells, leukocytes and NK cells, had 4.3-fold lower expression in fatal cases (Fig 2). We observed similar reductions (2.6–3.0-fold) in three chemokine receptor transcripts, *CX3CR1*, *CXCR3*, and *CCR3* (Fig 2). Fatal cases also had 2.0–5.0-fold lower abundance of six genes involved in blood coagulation, most notably platelet factor 4 (*PF4*/*CXCL4*), pro-platelet basic protein (*PPBP*/*CXCL7*), and Factor 13 (F13A1) (Fig 2). Together these data suggest that fatal cases had diminished migration of immune cells to sites of infection as well as reduced expression of coagulation factors, which could contribute to the hemorrhaging observed in many fatal leptospirosis cases.

We identified a prominent diminution in the abundance of Groups 1 and 2 transcripts involved in antigen presentation and the generation of an adaptive immune response in fatal cases (Figs 1C, 1D and 2; S2 and S4 Tables) including 2.1–3.9-fold reductions in the abundance of six HLA Class II transcripts and *CD74* (invariant chain). We observed reduced abundance of 10 transcripts involved in T cell activation and regulation in fatal cases such as 2.7 and 3.2-fold decreased abundance of *CD40LG*, a T cell protein, which promotes immunoglobulin class switching, and *CD27*, important for T and B cell memory and immunoglobulin class switching (Fig 2). Further, we identified decreased abundance of 25 pathways related to B cell and antibody responses in fatal cases (S2 and S4 Tables), with a 2.8- to 13.6-fold decreased expression for 47 immunoglobulin genes and reduced abundance of transcripts for B cell signaling (*BLNK*), IgM production (*MZB1*), and germinal center formation (*POU2AF10*) (Fig 2). These results suggest that fatal cases may not be capable of mounting robust T cell and B cell responses during acute infection because of defects in antigen presentation. Adult patients with Gram negative septic shock also generate transcriptional profiles with reduced T cell activation and antigen presentation suggesting fatal leptospirosis cases may share clinical features with bacterial sepsis [35].

### Stronger Adaptive Immune Responses in Patients Lacking Acute Lung Injury

**Fig 3:**
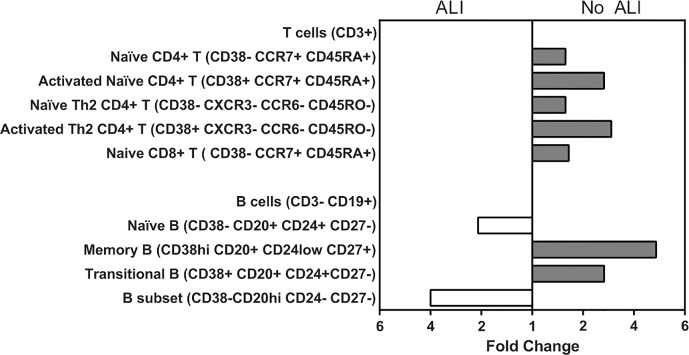
More robust T and B cell responses in patients lacking acute lung injury. PBMCs from patients with ALI (N = 4) and hospitalized patients lacking pulmonary complications (No ALI; N = 9) during acute leptospirosis. Cells were labeled with fluorescent antibodies for immunophenotyping and analyzed by flow cytometry [36]. Live CD3^+^ cells or CD3^–^/CD19^+^ cells were sampled and clustered by Citrus analysis, based on the expression of markers in each panel. Abundance of subsets was compared using SAM (FDR < 5%) between No ALI and ALI groups. Data shown represent fold change ratios of cell abundance for the indicated cell subsets.

To examine whether patients with severe infection had diminished adaptive immune cell activation or frequencies, we employed multi-parameter flow cytometry of peripheral blood mononuclear cells (PBMCs) to profile T cell and B cell responses in 11/13 survivors and 1/3 deaths (Fig 3). Acute lung injury (ALI, defined in Materials and Methods) is a significant risk factor for death in leptospirosis [8, 17]. Because we had limited PBMCs from deceased patients, we stratified patients by ALI to distinguish cases with higher probabilities of fatality. The ALI group had significantly fewer CD4^+^ and CD8^+^ T cells and larger percentages of naïve B cells [36]. In contrast, the patient group lacking pulmonary complications (No ALI) had elevated memory B cell and transitional B cell populations, the subsets required for antibody production [36]. Both the B and T cell subsets associated with immune activation and memory were lower in the more severe ALI group, which included one fatal case (Fig 3) [36]. These phenotypic changes are consistent with our microarray findings and suggest that fatal cases had dampened adaptive immune responses in the peripheral blood.

### Lower Antibody Titers and Elevated Bacterial Loads in Fatal Cases Are Consistent with Reduced Humoral Transcriptional Responses

Because we observed a significant reduction in transcription of immunoglobulin-encoding genes in fatal cases and reduced B and T cell responses in more severe disease, we quantified anti-*Leptospira* agglutinating antibodies in corresponding sera from the 16 patients with microarray results and an additional 18 fatal cases (N = 21 total) and 11 survivors (N = 24). Notably, we found that anti-*Leptospira* antibody titers were significantly lower in fatal cases (Tables 1 and S6). This is consistent with a decreased abundance of immunoglobulin transcripts (Fig 1B–1D). We also identified a significant correlation between levels of transcription of 21 immunoglobulin genes and agglutinating antibody titers during early acute infections, indicating a direct association between transcript levels and antibody titers (S5 Table). Further, the higher systemic bacterial loads detected in fatal cases inversely correlated with both immunoglobulin gene transcripts and antibody titers (β = -0.3811 ± 0.1554, *P* = 0.0188), providing functional data suggesting a critical role for decreased humoral responses in fatal cases.

### Fatal Cases Show Decreased Expression of Cathelicidin, an Antimicrobial Peptide, and Elevated Transcription of Proinflammatory Cytokine Pathways

The transcript with the greatest difference in abundance (17.6-fold) between nonfatal and fatal cases encodes an antimicrobial peptide (AMP), cathelicidin (CAMP) (Fig 2 and S3 Table). In survivors, cathelicidin had 20.3-fold higher expression during acute disease compared to convalescence (S1 Table). Interestingly, we found no association between disease outcome and other antimicrobial molecules produced by innate immune cells such as resistins, defensins, or elastase, although we detected increased abundance of these transcripts in acute illness relative to convalescence in survivors (S1 Table). Therefore, cathelicidin is the only antimicrobial peptide with significantly decreased expression in fatal cases.

In addition to cathelicidin, fatal cases had many transcripts with significantly elevated expression compared to survivors (Group 3; 221/389), including two GO terms, “Interleukin 1 Receptor Activity” and “Sulfur Compound Biosynthetic Processes”, and two related functional pathways “IL-1 Signaling” and “Metabolism” (S2 and S4 Tables). Concordantly, we measured large relative increases in expression (2.9–9.4-fold) for the decoy IL-1 receptor (*IL-1R2*), the IL-1 receptor (*IL-1R1*), and IL-18 receptor (*IL-18R*), indicating transcription of these proinflammatory pathways may be relevant to outcome in fatal cases (Fig 2). We identified increased abundance of transcripts involved in NF-κB signaling, a pathway important for proinflammatory responses: *MKK3* and *MKK6*, members of p38 signaling pathways that respond to environmental stress. Additionally, we found elevated transcript levels of human growth factor (*HGF*), a gene induced by proinflammatory cytokines, although this may be in response to signaling or driven by higher bacterial loads [37, 38]. Together, these data suggest that the increased abundance of specific proinflammatory responses in nonsurvivors may have contributed to fatality.

### Serum Levels of Cathelicidin and IL-18 Differ Based on Disease Outcome

**Fig 4:**
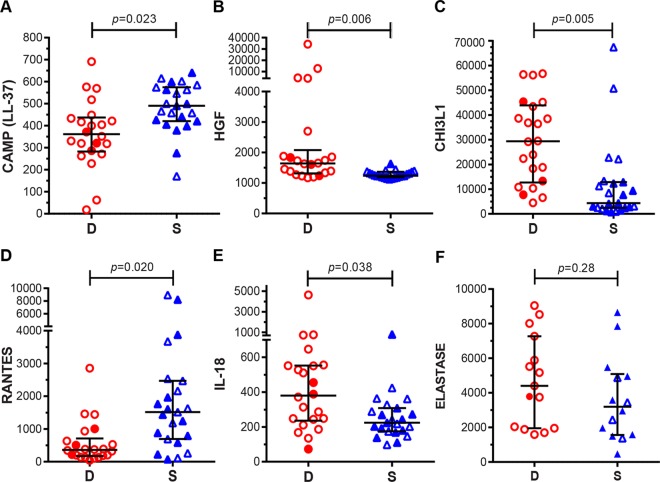
Serum protein levels validate expression profiles of specific gene products identified by microarray. Serum protein levels of cathelicidin (CAMP [LL-37]) **(A)** and RANTES **(D)** were higher in survivors, while HGF **(B)** and IL-18 **(E)** serum levels were higher in deceased patients. **(C)** Deceased patients had higher serum of CHI3L1 than survivors, in contrast to microarray results. **(F)** Elastase levels did not differ between outcomes. N = 23 for Survivors (S; blue triangles) and N = 22 for and deceased patients (D; red circles) for **(A-E)**. N = 14 for survivors and N = 15 for deceased patients in **(F)**. Filled symbols denote individuals included in microarray analyses. Values are medians +/- IQR in pg/mL **(B-E)** or ng/mL **(A, F)**.

The transcriptional studies identified more than 30 genes with striking differences between survivors and non-survivors, which may shed light on pathogenesis or have potential as new therapeutic targets or diagnostic markers (Fig 2). To investigate some of these targets, we quantitated serum levels of LL-37 (active fragment of cathelicidin, CAMP), IL-18, RANTES, HGF, and CHI3L1 by single or multiplex ELISA. (Fig 4A–4F; N = 45 patients, 22 of whom died during acute infection). Notably, we measured significantly higher serum levels of LL-37 in survivors, consistent with microarray findings, while finding no differences in the levels of elastase, an enzyme produced by neutrophils, suggesting some normal neutrophil function. Consistent with our microarrays, we found elevated serum protein levels of RANTES in survivors, and lower levels of HGF and IL-18, the ligand for IL-18R. These data provide further evidence that these genes and their products may play critical roles in disease progression. Lastly, levels of CHI3L1 protein were lower in survivors than in fatal cases (Fig 4). We do not know why these findings for CHI3L1 contrast with the results of gene expression analyses; however, lower CHI3L1 in surviving patients is consistent with its presence as a biomarker of severity in other inflammatory diseases [39].

### Risk Factors for High Bacteremia and Fatality in Leptospirosis

To identify factors associated with case fatality including the clinical, transcriptional, cell subset, and serum factors assessed in stratified leptospirosis patients, we employed univariate analyses of data for the entire patient cohort (S6 Table). As we noted in our initial cohort assessed for transcriptional analysis (Table 1), fatal cases had lower platelet counts, lower antibody titers, and higher bacterial loads. We also found an association of ALI with death. In contrast, survivors had less evidence of renal failure as measured by significantly lower maximum blood urea nitrogen, creatinine, and potassium levels, fewer hemodialysis treatments, and lower incidence of oliguria or anuria (S6 Table).

To identify independent risk factors for higher bacterial loads and death, we included all significant univariate variables (Fig 4) and days of symptoms in multivariate linear and logistic regression models, respectively (S1 Text). These analyses revealed that survivors had significantly higher titers of agglutinating antibodies (β = -0.3811 ± 0.1554; *P* = 0.02), and further, that lower serum levels of cathelicidin (LL-37) predicted higher bacterial loads (Table 2). Additionally, lower RANTES levels and higher CHI3L1 serum levels were independent risk factors for death in patients with leptospirosis (Table 2). Gender (*P* = 0.17), age (*P* = 0.10), and days of symptoms (*P* = 0.09), possible confounders of disease outcome, were not significantly different between the two patient groups.

**Table 2:** Risk factors associated with death in leptospirosis patients.

	Bacterial load (log)^a^				Death^b^			
Variable	Univariate^c^		Multivariate^d^		Univariate^e^		Multivariate^f^	
	β ± SE	P-value	β ± SE	P-value	β ± SE	P-value	β ± SE	P-value
Days of Symptoms^g^	-0.2041 ± 0.1334	0.134	-0.2404 ±0.1225	0.057	-0.0493 ± 0.141	0.727	0.5269 ± 0.3077	0.087
LL-37 (ng/mL)	-5.033E^-3^ ± 1.883E^-3^	**0.011**	-5.395E^-3^ ± 1.832E^-3^	**0.006**	-5.988E^-3^ ± 2.635E^-3^	**0.023**	-4.663E^-3^ ± 3.085E^-3^	0.131
IL-18 (pg/mL)	2.028E^-4^ ± 4.389E^-4^	0.647	-	-	4.247E^-3^ ± 2.043E^-3^	**0.038**	-	-
RANTES (pg/mL)	-1.994E^-4^ ± 1.563E^-4^	0.210	-	-	-9.764E^-4^ ± 4.189E^-4^	**0.020**	-1.345E^-3^ ± 6.540E^-4^	**0.040**
CHI3L1 (pg/mL)	3.697E^-5^ ± 1.466E^-5^	**0.016**	-	-	6.297E^-5^ ± 2.266E^-5^	**0.005**	6.590E^-5^ ± 2.787E^-5^	**0.018**
HGF (pg/mL)	4.901E^-5^ ± 5.917E^-5^	0.413	-	-	6.114E^-3^ ± 2.242E^-3^	**0.006**	-	-

^a^ Number of *Leptospira* genome equivalents/mL whole blood was the outcome for the univariate and multivariate analyses.

^b^ Death was the outcome for the univariate and multivariate analyses for acute, confirmed leptospirosis.

^c^ Univariate linear regression of each variable predicting the number of *Leptospira* genome equivalents/mL whole blood.

^d^ Final multivariate linear regression model (lowest AIC score using deletion method) predicting the number of *Leptospira* genome equivalents/mL of whole blood.

^e^ Univariate logistic regression of each variable predicting death from acute leptospirosis.

^f^ Final multivariate logistic regression model (lowest AIC score using deletion method). We excluded HGF due to nonlinearity of features.

^g^ Days of symptoms prior to blood collection.

### Cathelicidin Protects Against Lethal *Leptospira* Challenge in Hamsters

**Fig 5:**
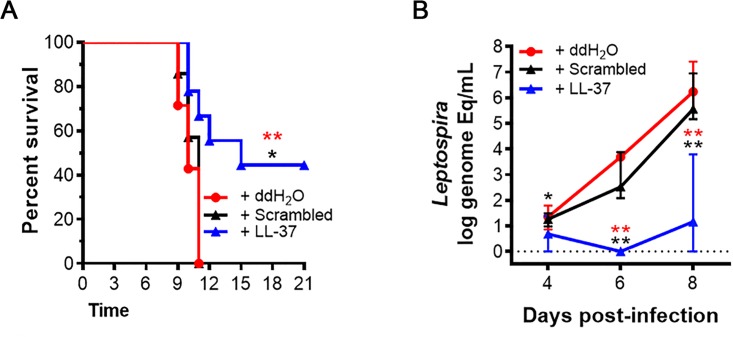
Cathelicidin (LL-37) protects hamsters from lethal *Leptospira* infection. (**A**) Survival in hamsters pre-treated with 1 mg/kg of cathelicidin (LL-37) (n = 7) was significantly greater than LL-37 scrambled peptide (Scrambled) (n = 7) (*P* = 0.016) or ddH_2_O-treated controls (ddH_2_O) (n = 7) (*P* = 0.008) following lethal challenge with 100 *Leptospira*. **(B)** Bacterial loads (*Leptospira* genome equivalents per mL of whole blood) in 7 infected hamsters were significantly lower at 4 (*P* = 0.035; *P* = 0.146), 6 (*P* = 0.003; *P* = 0.001), and 8 days (*P* = 0.003; *P* = 0.001) post-infection in LL-37-treated hamsters relative to 7 scrambled peptide (black) or ddH_2_O-treated controls (red), respectively. Shown are medians ± IQR. An * signifies a *P*-value ≤0.05; ** signifies *P*<0.01 as determined by Mantel Cox test for (**A**) or Mann-Whitney test for (**B**).

As our results suggest a critical role for cathelicidin during infection with *Leptospira spp*, we tested the effect of LL-37, the active peptide of cathelicidin, in a hamster model of lethal leptospirosis. Immediately prior to lethal infection with 100 live *Leptospira interrogans* serovar Copenhageni, we injected hamsters with LL-37 reconstituted in ddH_2_O, an LL-37 scrambled peptide reconstituted in ddH_2_O (control group), or water alone (control group). We found that while all hamsters in both control groups (N = 14) died within 11 days of infection with high blood bacteremia, hamsters treated with LL-37 (N = 7) were significantly protected from lethal infection, and controlled systemic bacterial loads (Figs 5 and S3). These data provide strong evidence that cathelicidin is a critical immune molecule protecting against fatal leptospirosis.

## Discussion

Despite the important global disease burden of leptospirosis [3, 4], there are key gaps in our understanding of host pathogenic mechanisms that contribute to poor disease outcomes such as massive pulmonary hemorrhage and death. To identify host factors contributing to fatality, we conducted an in-depth characterization of clinical, transcriptional, immune cell subset, and serum factors in hospitalized leptospirosis patients, including the first comprehensive human transcriptome analysis of peripheral blood during acute leptospirosis. We demonstrated that low serum levels of cathelicidin (LL-37) is a risk factor for high bacterial loads and suggests cathelicidin is a novel, potential therapeutic for leptospirosis. Additionally, we identified CHI3L1 and RANTES, as new risk factors for death from leptospirosis. Our data suggests a lower magnitude of specific innate immune responses may underlie poor early control of infection and diminished activation of adaptive immune responses. Subsequently, increased bacterial proliferation promotes systemic inflammation, contributing eventually to patient death. The mechanistic details of this proposed model of pathogenesis remain to be determined.

The most pronounced finding in the transcriptional profiling was the markedly lower level of transcripts encoding the antimicrobial peptide, cathelicidin, in fatal cases. The defect in production of antimicrobial peptides was not a global innate immune dysfunction, as we found no significant differences in other antimicrobial transcripts or serum proteins (elastase, resistins, and defensins) between survivors and fatal cases. We identified differences in abundances in only two Toll-like Receptors: TLR8 (can detect single-stranded bacterial RNA) [40, 41], which was elevated in fatal cases likely due to higher bacterial loads, and TLR7 (senses bacterial RNA in phagosomes) [42, 43], which was less abundant in fatal cases, possibly due to fewer phagocytic cells.

Cathelicidin functions as an antimicrobial peptide, capable of directly killing bacteria, fungi, parasites, and some viruses [37]. Consistent with our results, direct anti-leptospiricidal activity has been demonstrated for the active peptide of cathelicidin, LL-37, in vitro [44, 45]. Unlike other antimicrobial peptides, cathelicidin is also an important activator of neutrophils, stimulating phagocytosis, diminishing apoptosis, and reducing LPS-driven TLR-dependent proinflammatory responses [37]. Reduced levels of circulating cathelicidin therefore could contribute to elevated bacterial load, which we observed in the hamster model, and higher levels of proinflammatory cytokines, such as IL-1 and IL-18, which we observed in fatal human cases. Consistent with our current findings, others and we have shown previously that high levels of proinflammatory cytokines, and their transcripts, IL-1α, IL-6, and IL-8 as well as the IL-1 antagonist receptor 1, are associated with poor disease outcomes for leptospirosis [21, 23, 24, 46]. These results strongly suggest that decreased cathelicidin might contribute both to decreased bactericidal activity and increased levels of inflammation, resulting in greater tissue damage and higher bacterial loads. These findings, combined with our animal experiments and recent biochemical [47] and clinical studies [48, 49] involving cathelicidin, suggest a potential role for cathelicidin during acute illness as a novel therapeutic option for patients with leptospirosis.

We identified several markers of inflammation in fatal cases: CHI3L1, HGF, and proinflammatory cytokine receptors, IL-18R and IL-1R1. CHI3L1 expression is induced by proinflammatory cytokines, and is associated with increased patient mortality in sepsis and other infectious or inflammatory diseases [50]. Proinflammatory cytokines also induce expression of HGF, a pleiotropic cytokine, which decreases inflammation, inhibits antigen presentation, and promotes organ injury repair [38]. HGF promoted healing in a mouse model of lung injury, and is in early clinical trials for reducing inflammation in acute spinal cord injuries [38]. We detected higher levels of HGF in fatal cases, suggesting these patients had greater systemic inflammation than survivors. IL-1 and IL-18 are cytokines produced following TLR signaling and inflammasome activation to induce downstream immune responses and inflammation [51]. Patients with poor disease outcomes from other critical illness, such as sepsis, also have elevated levels of IL-18 [13, 52, 53]. Several clinical trials are assessing the efficacy of IL-18 inhibition in primarily chronic inflammatory diseases, but their application to leptospirosis will require consideration of potential protective roles for IL-18. Together, these data suggest CHI3L1, IL-18, and HGF represent new potential prognostic and therapeutic strategies for leptospirosis.

Our study illustrated the importance of the adaptive immune response, and in particular the antibody response, in protection from fatal leptospirosis. While the humoral immune response is accepted widely as the primary mode of immunity to *Leptospira* infection, a protective role for antibodies has not been demonstrated definitively in humans. Passive transfer experiments in animal models of leptospirosis have shown that anti-LPS antibodies confer protection from homologous reinfection [54, 55]. In keeping with these data, we detected significantly lower antibody titers and transcript abundance for immunoglobulins in patients that did not survive. The notable decrease in chemokines, such as RANTES, which functions to recruit immune cells to sites of infection, and which we identified as a risk factor for death, suggests aberrant cell trafficking could contribute to poor or slower adaptive immune response generation in fatal cases. However, further studies are needed to determine the mechanistic causes of neutropenia and lymphocytopenia in fatal cases, despite lower LL-37 and chemokine levels. Lastly, we observed a larger number of memory B cell and transitional B cell responses in patients with less severe leptospirosis, raising the intriguing idea that more severe disease may represent primary infection and that secondary infections, where some memory B cell responses are available for recall, may be less severe. Taken together, our data support the animal data in which anti-*Leptospira* antibodies are critical for bacterial clearance and improved disease outcomes [12, 56, 57].

The associations we identified in our microarray findings are strengthened by the functional assays we performed on the larger cohort of confirmed patients and the animal studies. However, our patients represent primarily individuals of mixed Caucasian and African descent and it will be important to identify whether the pathways we identified are generalizable to global populations, given that several studies have shown association of specific alleles with increased susceptibility to leptospirosis [15, 16, 58, 59]. Further, it will be important to compare our findings on whole blood transcriptional profile with samples from the lungs in patients that develop LPHS. Studies of the specific tissue site may reveal additional immune dysfunction in the lungs.

Our study provides the first evidence that patients die from leptospirosis because of a failure to mount innate and adaptive immune response to this pathogen. While we were able to analyze only a small number of patients, the results demonstrate the power of using systems biology approaches to understand disease pathology. We have identified several unique targets, which may represent new diagnostic and treatment of leptospirosis patients at greatest risk of death. CHI3L1 and RANTES serum levels are attractive candidate diagnostic markers, which could identify patients at risk for developing severe disease and allow hospitals to focus limited resources on patients with greatest risk. Most importantly, the development of anti-*Leptospira* antibody therapies or administration of cathelicidin are potential new strategies for reducing bacterial loads in severely ill patients.

## Methods

### Ethics Statement

The Yale Institutional Review Board (HIC#1006006956), the Ethics Committees at Fiocruz-Salvador (CEP-CPqGM 329) and Hospital Couto Maia (175), and the Brazilian Ministry of Health National Ethics Committee in Research (CONEP 15925) approved the study protocol prior to study initiation. Our trained study team obtained written informed consent in the native language (Portuguese) from all participants prior to blood and data collection.

All animal protocols and work were approved and conducted under the guidelines of the Yale Institutional Animal Care and Use Committee (IACUC), under approved protocol #2014–11424. The Yale IACUC strictly adheres to all Federal and State regulations, including the Animal Welfare Act, those specified by Public Health Service, and the US Department of Agriculture, and uses the *US Government Principles for the Utilization and Care of Vertebrate Animals Used in Testing*, *Research*, *and Training* as a guide for all animal studies.

### Study Design

We performed active surveillance at an infectious disease hospital in Salvador, Brazil, to identify patients with suspected leptospirosis between April 2013 and September 2013 with the goal of discovering markers associated with case fatalities. We used previously described criteria to identify cases: <15 days of fever, jaundice, high serum creatinine and/or blood urea nitrogen, acute lung injury ([ALI]; defined by mechanical ventilation, ≥250 mL blood in lungs or endotracheal tube, and/or respiration rate >38/min), oliguria (<500 mL urine/24 h), and epidemiologic data supporting likelihood of exposure to *Leptospira spp* [17]. For transcription studies, we stratified patients by survival, and for immunophenotyping by ALI [17]. We confirmed cases using serum microagglutination test (MAT) (13/16), qPCR (*Leptospira* genome/mL blood) (5/16), and/or blood culture (2/16), as described previously [7, 17, 18, 60, 61]. We collected clinical data during patient interviews and from hospital charts for all enrolled patients using a RedCap database [62, 63]. In surviving patients, we collected two venous blood samples: acute phase (≤72h of hospital admission; one patient collected at 168h; mean collection time: survivors 8.4 ±1.9d, fatal cases 6.7±2.3d) and convalescence (32-90d post-admission). We collected the identical acute sample from fatal cases, and a sample from four healthy individuals with prior *Leptospira* exposure (303-367d post-admission). We collected whole blood directly into red-top tubes (sera for ELISAs, MATs, and MSDs), PAXgene solution (RNA microarrays), CPT tubes (peripheral blood mononuclear cells [PBMCs]), EDTA tubes (qPCR), or EMJH culture medium (blood culture), processed and froze all samples at -70°C the same day of collection. We bar-coded all samples, monitored transport temperature, and recorded all cold chain data including sample receipt, processing time, and freezing time.

### Microarray Data and Analysis

#### Microarray sample preparation

We extracted RNA from thawed PAXgene samples using a Qiagen PAXgene Blood Kit according to the manufacturer’s protocol (Qiagen, Cat#762164). We used 22.5ng of total RNA (quality confirmed on Agilent Bioanalizer) from each sample, or the RNA spike in controls (Agilent; 10 x 32 E1A spike-in control probes), for initial cDNA synthesis. We prepared and purified Cy3-labeled cRNA using the Low Input QuickAmp Labeling Kit One-Color (Agilent, Cat#5190–2305) and RNA Spike-In Kit, One-Color (Agilent, Cat#5188–5282). We fragmented and hybridized 600ng of Cy3-labeled cRNA to SurePrint G3 Human Gene Expression 8x60K v2 Microarrays according to manufacturer’s specifications (Agilent, Cat#G4851B; 50,599 biological features).

#### Microarray scanning and data processing

We scanned the microarrays using the Agilent Microarray Scanner (Agilent Technologies), and collected data using Agilent Feature Extraction Software (v10.7). We quantile normalized data using limma, and retained data in all instances in which the signal was >64 in at least 3 samples [64]. We averaged the signal from replicate probes. All data are available at the NCBI Gene Expression Omnibus, accessible through GEO Series accession number GSE72946 (http://www.ncbi.nlm.nih.gov/geo/query/acc.cgi?acc=GSE72946) [65].

#### Microarray data analysis

We used Significant Analysis of Microarrays (SAM) to identify differentially expressed probes [66]. Unless otherwise stated, differences were considered significant if the false discovery rate (FDR) <1% and there was at least a 2-fold difference in the average expression between compared groups. We identified principal components using singular value decomposition [67]. We calculated Spearman’s correlation coefficients between relative gene expression levels and clinical variables using a perl-based script after deriving a null distribution through permutation of label levels [68].

For functional annotation of specific groups of transcripts, we employed the Database for Annotation, Visualization, and Integrated Discovery and Innate DB [33, 34, 69]. We considered DAVID functional categories (GO terms) significant if the FDR <0.01 and the p<0.05 after Benjamini correction for multiple hypothesis testing. We selected only the GO term with the smallest p-value for each significant DAVID cluster to avoid identifying redundant categories. We listed all identified GO terms in S3 Table. We performed subset analyses using the recommended hypergeometric analysis algorithm and Benjamini Hochberg p-value correction for Pathway, Transcriptional Factor, and GO term analyses (S4 Table). We considered results with corrected p<0.05 significant.

### Flow Cytometry and Clustering Analysis

We isolated peripheral blood mononuclear cells (PBMCs) from the blood of leptospirosis patients using CPT tubes and cryopreserved them in 90% FBS containing 10% DMSO and stored in liquid nitrogen until batch analysis as described [70]. On the day of analysis, we thawed cells and labeled them with fluorescent antibodies for immunophenotyping as follows: 1) T cell panel: HLA-DR, CD38, CD28, CD8, CCR7, CD45RA, CD27, and CD4; 2) T_H_1/2/17 cell panel: CD4, CD38, CD45RO, CD8, CXCR3, CCR6, CXCR5, and CCR4; 3) T_reg_ cell panel: HLA-DR, CD127, Foxp3, CD45RO, CD25, CCR4, CD39, and CD4; and 4) B cell panel: IgD, CD38, CD20, CD24, CD27, and CD10 [36]. We analyzed cells by flow cytometry using a custom, programmed BioMek robotic platform and detected using an LSR Fortessa (BD BioSciences) [70].

We employed two-dimensional gating analysis of flow cytometry files by FlowJo (Treestar) to remove doublets and debris using scatter channels. We labeled living cells with a viability marker and pre-gated for T cells (CD3^+^) or B cells (CD3^-^). Immunophenotyping panels defined T cell subsets (T_H_1/2/17 cell, and T_reg_) or B cells (CD3^–^CD19^+^). We clustered cell subsets as defined above using Citrus version 0.08 (https://github.com/nolanlab/citrus) to compare no ALI and ALI (met criteria for ALI described above and/or died) samples [71]. The SAM model type employed file sample size of 200 events, and the minimum cluster size was <5%, significance for false discovery rate (FDR) (*q* < 0.05). We performed each comparison at least 3 times to ensure reproducibility [71].

### ELISAs for LL-37, Elastase, IL-18, CHI3L1, HGF, and CCL5 Meso Scale Discovery (MSD) Assays

We quantified the levels of LL-37, the active peptide form of cathelicidin (HyCult Biotech, Cat#HK321-02), and elastase (Hycult Biotech, Cat#HK319) by ELISA using duplicate dilutions of sera collected from the patients described in this study, and sera frozen at -80°C from an additional 33 patients (49 total) with laboratory-confirmed leptospirosis: 13 survivors (25 total) and 21 nonsurvivors (24 total). Due to sera availability, we measured elastase in only 29 patients: 14 survivors and 15 deceased patients. We measured serum levels of IL-18, CHI3L1, HGF, and CCL5 using technical replicates on single-plex MSD kits for each molecule as specified by the manufacturer (Meso Scale Discovery, IL-18: K151MCD-1; RANTES Ultra-Sensitive Kit: K151BFC-1; HGF: K151HDC-1; and CHI3L1/YKL-40: K151NHD-1).

### Hamster Infection Model

We intraperitoneally infected 3-week old Golden Syrian hamsters with 100 live leptospires (*Leptospira interrogans* serovar Copenhageni strain Fiocruz L1-130) immediately following intracardiac injection of 1 mg/kg LL-37 (synthetic peptide) in ddH2O (BACHEM; treated group), 1 mg/kg scrambled LL-37 (scrambled control [BACHEM Cat. H-7886]), or the identical volume of ddH_2_O (ddH_2_O control group) [72, 73]. On days 4, 6, and 8 after infection, we performed qPCR on peripheral blood as described above. We monitored animals a minimum of two times daily. We immediately euthanized moribund or animals with signs of clinical disease by CO_2_ inhalation.

### Statistical Analysis

We used GraphPad Prism 6.0, R, and EpiInfo 7 to perform all statistical analyses except for microarray data, which we analyzed as described above. We performed descriptive statistics on continuous variables, and used the Fisher exact test or Mann-Whitney t-test to compare categorical or continuous variables, respectively, between survivor and deceased groups. We performed linear regression and logistic regressions in R, using backward elimination, to predict bacterial load and death, respectively. For the multivariate regression predicting death, we used a backward elimination approach to identify the best model fit using variables that were significantly associated with death in univariate analysis and days of symptoms prior to blood collection. We did not include HGF in the logistic regression analysis due to a high number of outliers resulting in non-linearity of features (S2 Fig). We considered *P*<0.05 significant.

## Supporting Information

S1 Fig Differential gene expression between acute and convalescence in survivors with leptospirosis. (**A**) The abundance of these transcripts differs significantly between acute survivors (S) and healthy volunteers (H), but not between convalescent (C) and healthy samples. Rectangles denote transcript clusters with similar expression profiles and functions: green rectangles denote transcripts with higher abundance in S vs C or H and gray rectangles mark those with lower abundance. Also shown are days of reported symptoms prior to blood collection. (**B**) Significant GO Terms for transcripts with higher abundance in S vs C, and (**C**) transcripts with higher abundance in S vs C. **(D)** Scatter plot of log_2_ fold-change of significant transcripts for deceased (D) vs S (red) overlaid with those shared with S vs C (black). Zero indicates no change, while negative numbers indicate the transcripts for survivors in D vs S or at the convalescent time point (C) were elevated relative to deceased patients or acute phase, respectively.
(TIF)

S2 Fig Determining model fitness for experimental variables associated with death. In order to assess the linearity of features and goodness of model fit (blue lines), we plotted the observed values of variables associated with death (x-axis) as an outcome for confirmed leptospirosis cases versus the predictive probability of death (y-axis) within a 95% confidence interval (dotted or solid black lines). Modeling is described in the Supplemental Methods.
(TIF)

S3 Fig Cathelicidin (LL-37) protects hamsters from lethal *Leptospira* infection. (**A**) Survival in hamsters pre-treated with 1 mg/kg of cathelicidin (LL-37) (n = 14) was significantly greater than ddH_2_O-treated controls (n = 14) following lethal challenge with 100 *Leptospira* (*P*<0.0001). **(B)** Bacterial loads (*Leptospira* genome equivalents per mL of whole blood) in 14 infected hamsters were significantly lower at 4 (*P* = 0.010), 6 (*P* = 0.004), and 8 days (*P* = 0.0006) post-infection in LL-37-treated hamsters relative to 14 ddH_2_O-treated controls. Shown are medians ± IQR. An ** signifies a *P*-value ≤0.01; ***, *P*<0.001; and ****, *P*<0.0001 as determined by Mantel Cox test for (**A**) or Mann-Whitney test for (**B**).
(EPS)

S1 Table Differentially expressed transcripts in Acute vs Convalescent Survivors (SvC). (XLSX)

S2 Table All significant Functional GO terms for DE transcripts in Acute vs Convalescence (SvC) and Deceased vs Survivors (DvS). (XLSX)

S3 Table Differentially expressed transcripts in Deceased vs Survivors (DvS). (XLSX)

S4 Table All significant REACTOME functional pathways for differentially expressed transcripts in Deceased vs Survivors (DvS). (XLSX)

S5 Table Association between acute antibody titer and immunoglobulin transcript fold-change. (XLSX)

S6 Table Clinical signs and symptoms for patients with leptospirosis. (XLSX)

S1 Text Statistical modeling of possible risk factors predicting death. (DOCX)
