# Correction: Cathelicidin Insufficiency in Patients with Fatal Leptospirosis

**DOI:** 10.1371/journal.ppat.1006646

**Published:** 2017-09-26

**Authors:** Janet C. Lindow, Elsio A. Wunder, Stephen J. Popper, Jin-na Min, Praveen Mannam, Anup Srivastava, Yi Yao, Kathryn P. Hacker, Khadir Raddassi, Patty J. Lee, Ruth R. Montgomery, Albert C. Shaw, Jose E. Hagan, Guilherme C. Araújo, Nivison Nery, David A. Relman, Charles C. Kim, Mitermayer G. Reis, Albert I. Ko

The following information is missing from the Funding section: PJL was supported by the Flight Attendant Medical Research Institute (FAMRI).

## References

[1] Lindow JC, Wunder EA Jr, Popper SJ, Min J-n, Mannam P, Srivastava A, et al (2016) Cathelicidin Insufficiency in Patients with Fatal Leptospirosis. PLoS Pathog 12(11): e1005943 https://doi.org/10.1371/journal.ppat.1005943 27812211 10.1371/journal.ppat.1005943 PMC5094754

