# Supplementary figures and images for: Cathelicidin Insufficiency in Patients with Fatal Leptospirosis
Source: PLoS Pathog. 2016 Nov 3;12(11):e1005943. doi: 10.1371/journal.ppat.1005943 (PMC5094754; doi:10.1371/journal.ppat.1005943)

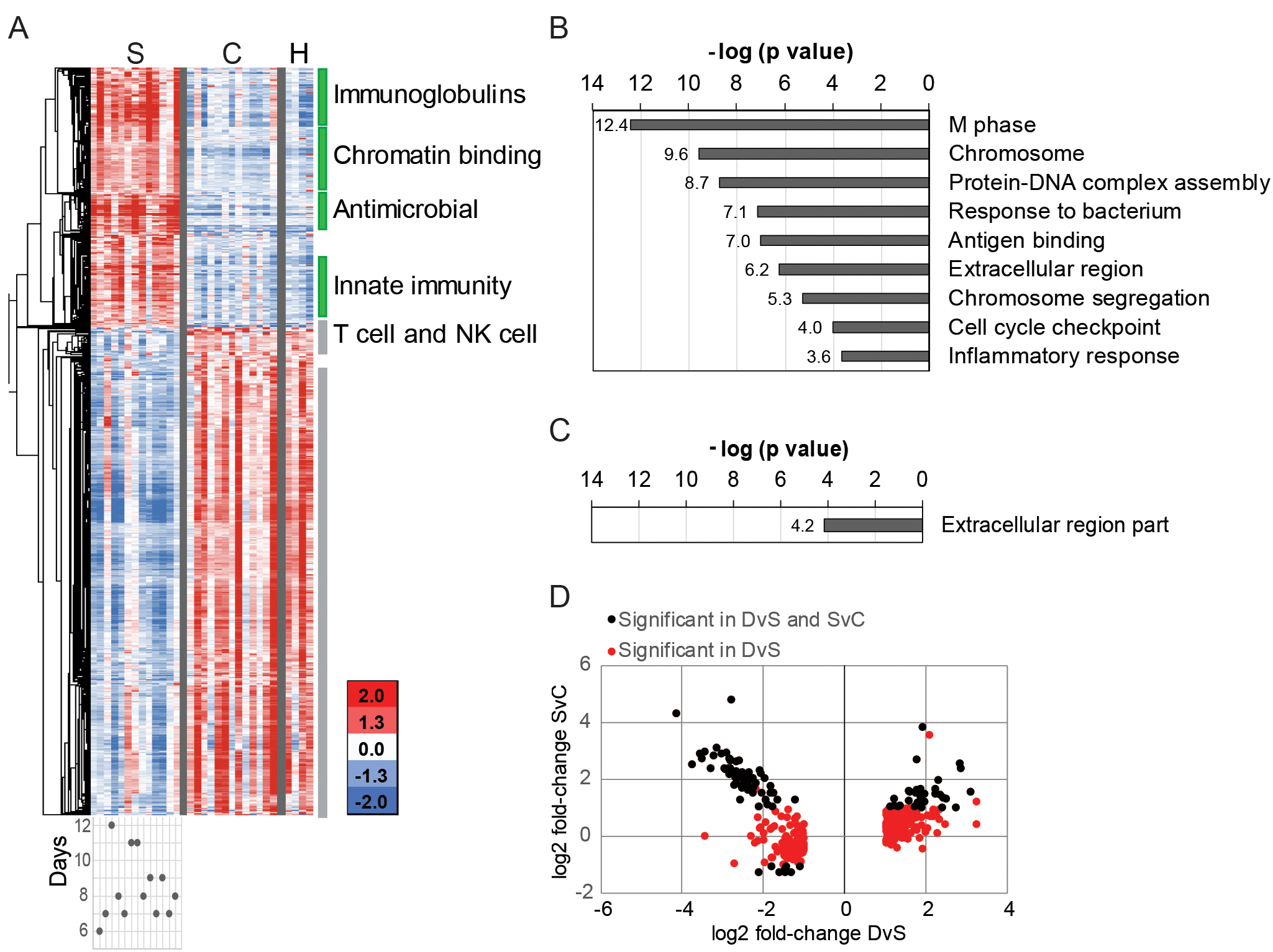

Supplement: S1 Fig — (A) The abundance of these transcripts differs significantly between acute survivors (S) and healthy volunteers (H), but not between convalescent (C) and healthy samples. Rectangles denote transcript clusters with similar expression profiles and functions: green rectangles denote transcripts with higher abundance in S vs C or H and gray rectangles mark those with lower abundance. Also shown are days of reported symptoms prior to blood collection. (B) Significant GO Terms for transcripts with higher abundance in S vs C, and (C) transcripts with higher abundance in S vs C. (D) Scatter plot of log2 fold-change of significant transcripts for deceased (D) vs S (red) overlaid with those shared with S vs C (black). Zero indicates no change, while negative numbers indicate the transcripts for survivors in D vs S or at the convalescent time point (C) were elevated relative to deceased patients or acute phase, respectively. (TIF) [file ppat.1005943.s001.tif]

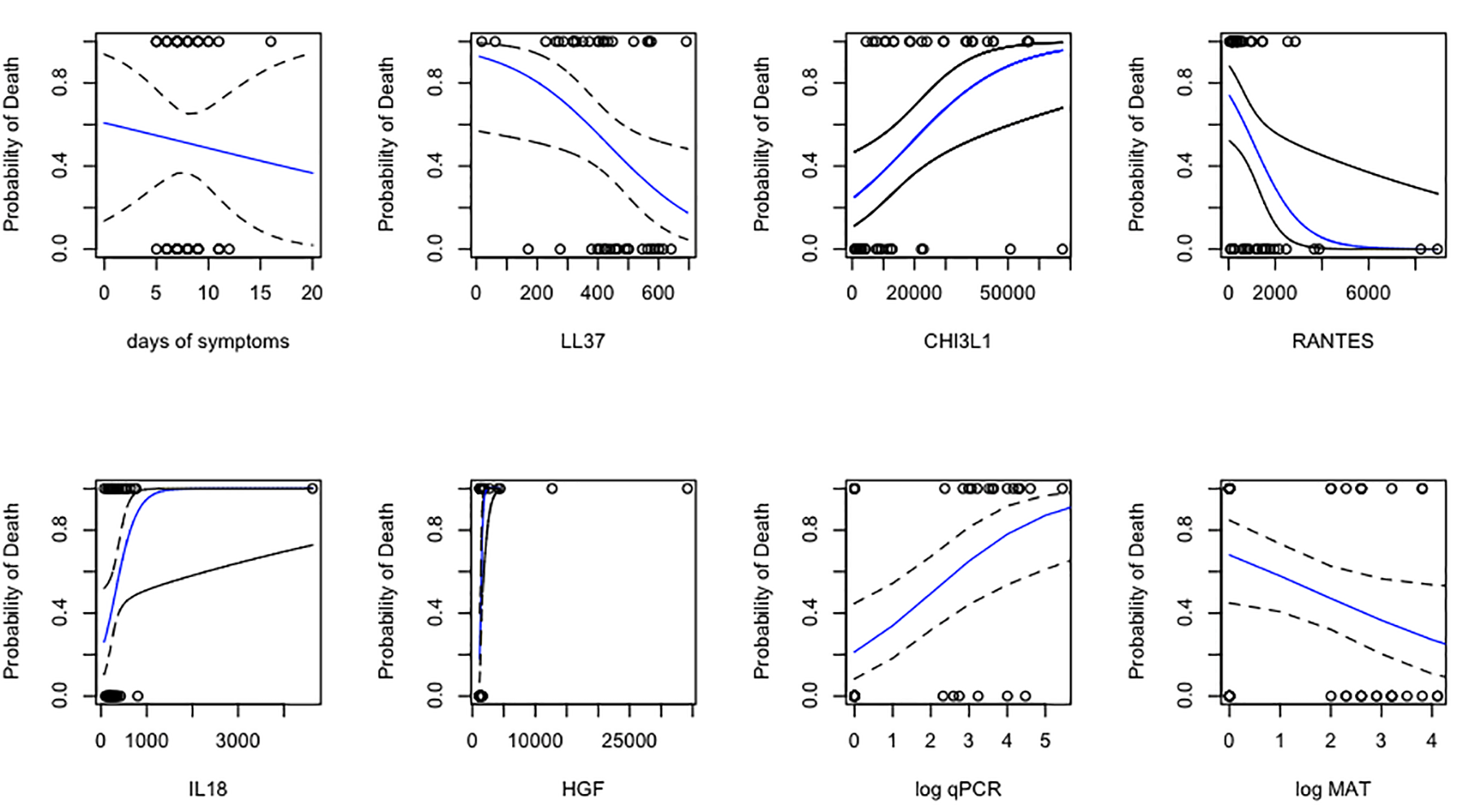

Supplement: S2 Fig — In order to assess the linearity of features and goodness of model fit (blue lines), we plotted the observed values of variables associated with death (x-axis) as an outcome for confirmed leptospirosis cases versus the predictive probability of death (y-axis) within a 95% confidence interval (dotted or solid black lines). Modeling is described in the Supplemental Methods. (TIF) [file ppat.1005943.s002.tif]
