# Supplementary material for: Cathelicidin Insufficiency in Patients with Fatal Leptospirosis
Source: PLoS Pathog. 2016 Nov 3;12(11):e1005943. doi: 10.1371/journal.ppat.1005943 (PMC5094754; doi:10.1371/journal.ppat.1005943)
Supplement: S1 Text — (DOCX) [file ppat.1005943.s010.docx]

**S1 Text Supplemental Methods**

**Microarray Data and Analysis**

We performed all preparations of microarray samples, experiments, and analyses, including functional analyses, as described in the Materials and Methods in the main text for the 13 paired acute and convalescent samples in confirmed leptospirosis patients and 4 healthy, Brazilian volunteers (healthy).

**Statistical Modeling to Identify Risk Factors**

As described in the main text, we used R software to perform the multivariate regression predicting death, using a backward elimination approach to identify the best model fit using variables that were significantly associated with death in univariate analysis and days of symptoms prior to blood collection. To assess the performance of each variable within the models generated, we plotted the variables associated with death against the predictive probability of death.
